# Generalized thymoma-associated myasthenia gravis associated with anti-α-amino-3-hydroxy-5-methyl-4 isoxazolepropionic acid receptor antibody encephalitis: A case report

**DOI:** 10.1097/MD.0000000000050330

**Published:** 2026-08-21

**Authors:** Jun Soma, Jun Sawada, Momoko Nanbu, Rei Ando, Shiori Kikuchi-Takeguchi, Keiko Tanaka, Naoki Nakagawa

**Affiliations:** a Division of Respiratory Medicine and Neurology, Department of Internal Medicine, Asahikawa Medical University, Asahikawa, Hokkaido, Japan; b Department of Multiple Sclerosis Therapeutics, Fukushima Medical University, School of Medicine, Fukushima, Fukushima, Japan; c Department of Animal Model Development, Brain Research Institute, Niigata University, Niigata, Niigata, Japan; d Division of Cardiology and Nephrology, Department of Internal Medicine, Asahikawa Medical University, Asahikawa, Hokkaido, Japan.

**Keywords:** anti-α-amino-3-hydroxy-5-methyl-4 isoxazolepropionic acid receptor antibody encephalitis, myasthenia gravis, thymoma

## Abstract

**Rationale:**

Autoimmune neurological disorders, including myasthenia gravis and autoimmune encephalitis, may occur in association with thymoma, warranting careful neurological surveillance. Generalized thymoma-associated myasthenia gravis (g-TAMG) complicated with anti-α-amino-3-hydroxy-5-methyl-4 isoxazolepropionic acid receptor (AMPAR) antibody encephalitis is rare, and its clinical features remain unclear.

**Patient concerns:**

A 53-year-old Japanese man was followed up at our department for g-TAMG. The thymoma was classified as B1/B2 mixed according to the World Health Organization classification. He initially developed insomnia, followed by progressive tremors in both upper limbs, dysphagia, and gait disturbance. He subsequently began to experience repeated falls.

**Diagnoses:**

Brain magnetic resonance imaging upon admission revealed hyperintense lesions in the extensive cerebral cortex on fluid-attenuated inversion recovery and diffusion-weighted imaging. During this course, serum and cerebrospinal fluid tested positive for AMPAR antibodies, leading to a diagnosis of AMPAR antibody encephalitis.

**Interventions:**

Intravenous methylprednisolone pulse therapy, intravenous immunoglobulin therapy, and plasma exchange therapy were administered, followed by rituximab.

**Outcomes:**

The neurological prognosis remained poor.

**Lessons:**

Immunotherapy with rituximab for generalized thymoma-associated myasthenia gravis (g-TAMG) associated with AMPAR antibody encephalitis showed no favorable outcomes. The poor prognosis may have been influenced by the fulminant encephalitis type and residual thymic tumor pathology. Although AMPAR antibody encephalitis is a rare complication of thymoma, its involvement should be considered when patients with thymoma develop central nervous system symptoms.

## 1. Introduction

Thymoma is associated with myasthenia gravis (MG) in 23% to 25% of patients.^[1,2]^ In addition to MG, thymoma has also been linked to other autoimmune neurological disorders, including autoimmune encephalitis.^[3–7]^ Therefore, careful monitoring for autoimmune complications is warranted in patients with thymoma.

This case report describes a patient with generalized thymoma-associated MG (g-TAMG) associated complicated by anti-α-amino-3-hydroxy-5-methyl-4 isoxazolepropionic acid receptor (AMPAR) antibody encephalitis treated with immunotherapy including rituximab.

## 2. Case report

**Figure 1.**
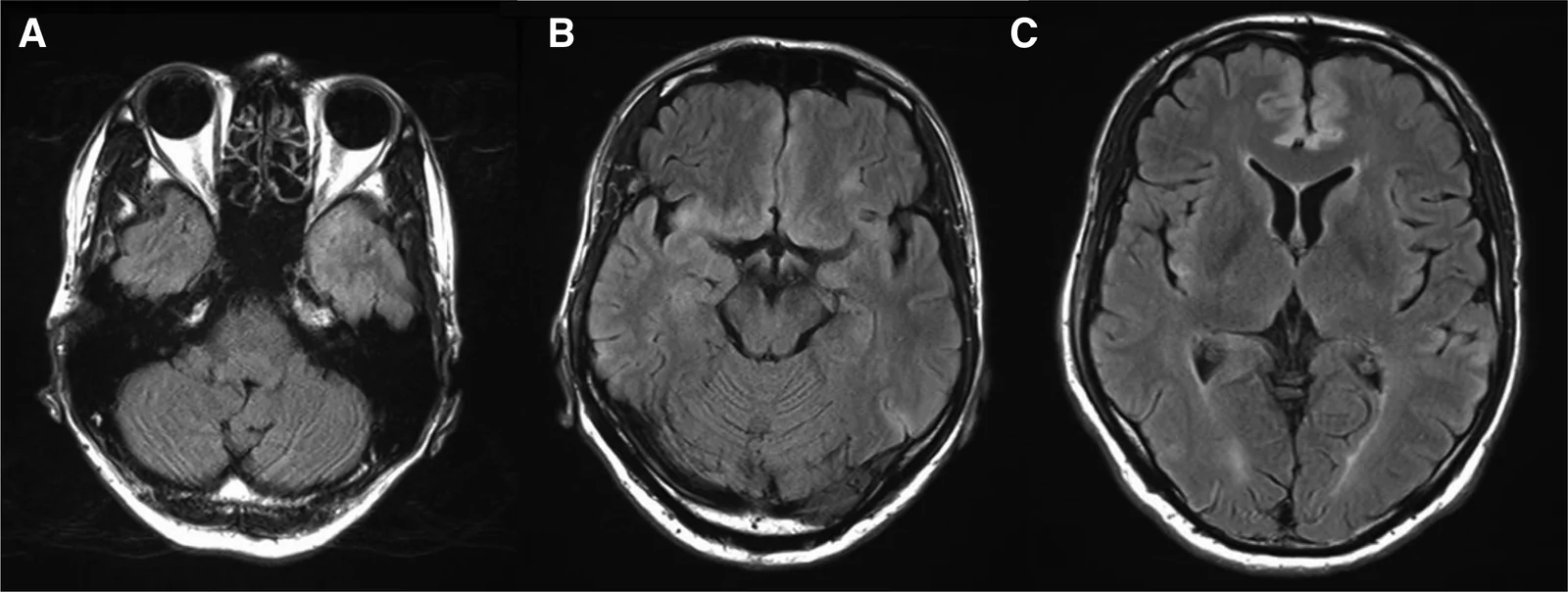
Brain magnetic resonance imaging on admission. Axial fluid-attenuated inversion recovery images (A–C) show hyperintense lesions in the extensive cerebral cortex.

**Figure 2.**
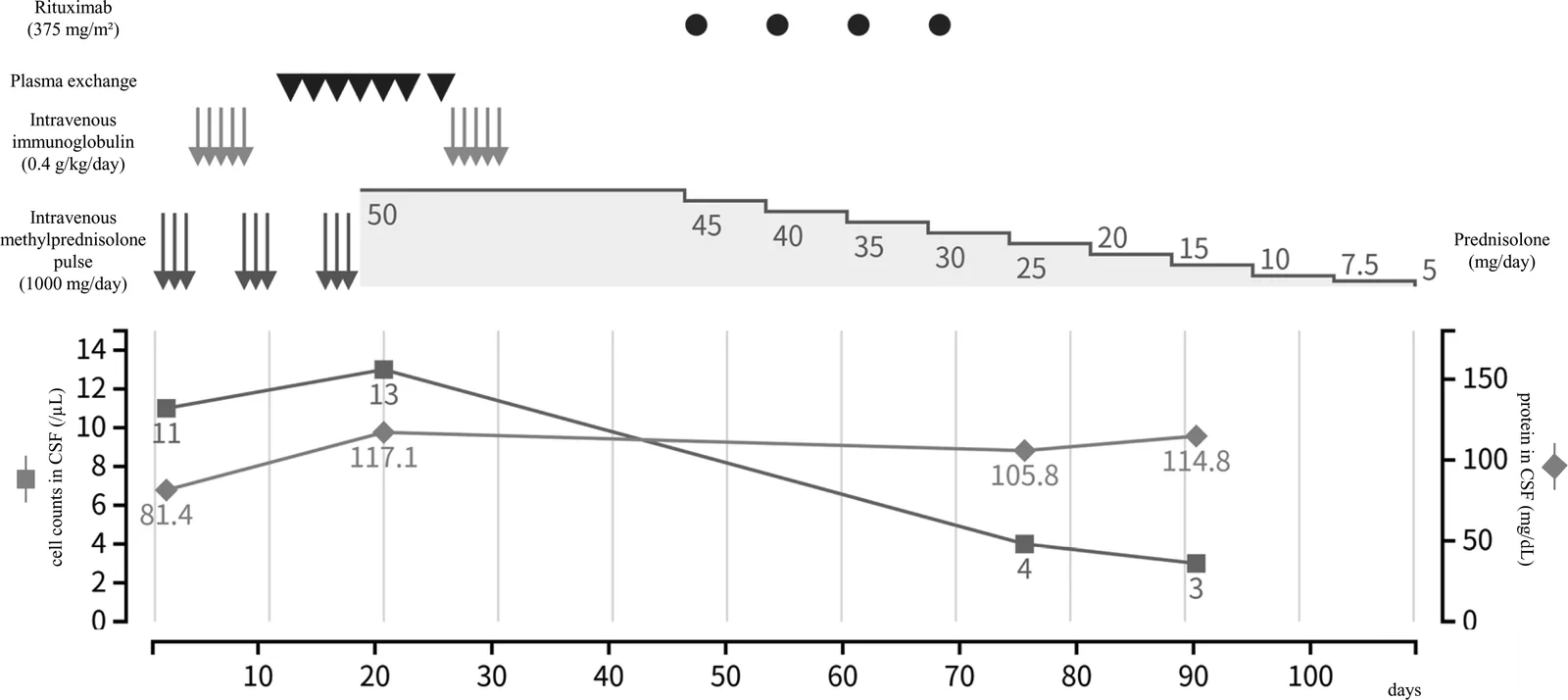
Timeline of cerebrospinal fluid cell counts and protein levels and immunotherapy. CSF = Cerebrospinal fluid.

**Figure 3.**
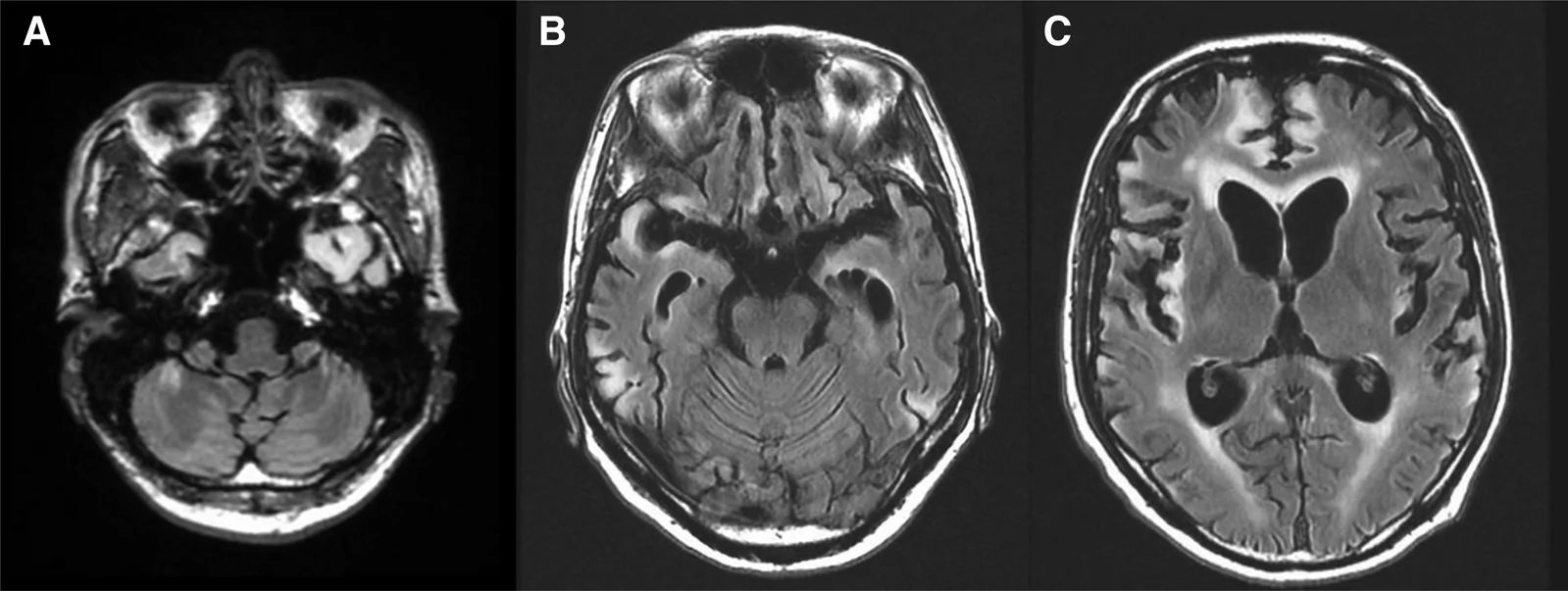
Brain magnetic resonance imaging on day 95 after onset. Axial fluid-attenuated inversion recovery images (A–C) demonstrate enlargement and increased conspicuity of cortical hyperintense lesions, with additional involvement of the insular cortex, cerebellum, and periventricular regions. Cerebral cortical atrophy and ventricular enlargement are also present.

The patient was a 53-year-old Japanese man. Eight months prior to presentation, he developed ptosis, diplopia, and fatigue. Generalized thymoma-associated myasthenia gravis (g-TAMG) was diagnosed based on a positive anti-acetylcholine receptor (AChR) antibody titer (84 nmol/L) and a positive Tensilon test. Following thymectomy, the patient’s symptoms improved, and he was subsequently followed without medication for g-TAMG. The resected thymoma was classified as World Health Organization B1/B2 mixed type with lymphofollicular hyperplasia. However, surgical margins were positive on pathological examination, and adjuvant radiotherapy was administered, which was completed one month prior to presentation. Ten days before admission, he developed insomnia, followed by progressive bilateral upper limb tremor, dysphagia, gait disturbance, and repeated falls. At presentation, he was mildly somnolent with slowed responses. No focal neurological deficits were identified, except for a postural tremor in both upper extremities, more pronounced on the right side. Routine laboratory investigations were within normal limits, including thyroid function and thyroid-associated, glutamate decarboxylase, and collagen disease-associated antibodies (Table 1). The serum anti-AChR antibody titer (84 nmol/L) remained essentially unchanged from the pre-thymectomy level to the time of admission. A paraneoplastic antibody immunoblot assay showed weak positivity for anti-titin and anti-Yo antibodies. Cerebrospinal fluid examination (CSF) revealed elevated mononuclear cell counts and protein, soluble interleukin-2 receptor, and interleukin-6 levels. Oligoclonal bands were negative. Autoantibodies against cell surface and synaptic proteins were examined in serum and CSF obtained prior to immunotherapy using a live-cell-based assay with HEK-293 cells transfected with AMPAR cDNA. Indirect immunofluorescence demonstrated positivity for AMPAR antibodies. Computed tomography (CT) showed no evidence of recurrent thymoma or other malignancy. Brain magnetic resonance imaging on admission revealed extensive cortical hyperintensities on fluid-attenuated inversion recovery sequences (Fig. 1). Intravenous methylprednisolone pulse therapy (1000 mg/day for 3 days) was initiated on the day of admission (Fig. 2). On the following day, the patient developed fever, hyperhidrosis, and paroxysmal sinus tachycardia. By day 4, neurological deterioration was evident, with worsening consciousness disturbance, generalized muscle rigidity, and limb myoclonus. Intravenous immunoglobulin therapy (0.4 mg/kg/day for 5 days) was initiated on the same day. Electroencephalography performed during myoclonus showed severe diffuse encephalopathy without paroxysmal epileptiform discharges. Given the presence of autonomic dysfunction and respiratory muscle fatigue, endotracheal intubation and mechanical ventilation were initiated on day 10, and plasma exchange therapy was subsequently performed. However, no neurological improvement was observed. On day 47, rituximab (375 mg/m^2^) was administered weekly for four doses, and prednisolone was tapered following the completion of three courses of iuntravenous methylprednisolone pulse. Although CSF pleocytosis decreased, elevated protein levels, impaired consciousness, and limb myoclonus persisted. Follow-up brain magnetic resonance imaging revealed progression of cortical hyperintensities involving the cerebral cortex, as well as new involvement of the insular cortex, cerebellum, and periventricular regions (Fig. 3). Cerebral cortical atrophy and ventricular enlargement were also noted. Given the poor neurological prognosis, the patient was transferred to a long-term care facility on day 125.

**Table 1:** Laboratory data at the time of admission.

Peripheral blood		Anti-AChR antibody (nmol/L)	79
White blood cells (3.30–8.60/µL)	6390	Anti-amphiphysin antibody	(–)
Red blood cells (4.35–5.55 × 10^4^/µL)	4.53	Anti-CV2/CRMP5 antibody	(–)
Hemoglobin (13.7–16.8 g/dL)	14.0	Anti-Ma2/Ta antibody	(–)
Hematocrit (40.7–50.1%)	40.5	Anti-Ri antibody	(–)
Platelet (15.8–34.8 × 10^4^/µL)	18.9	Anti-Yo antibody	(1+)
		Anti-Hu antibody	(–)
Coagulation study		Anti-recoverin antibody	(–)
PTINR (0.80–1.20)	0.99	Anti-SOX1 antibody	(–)
APTT (27.0–39.9 s)	28.2	Anti-titin antibody	(1+)
Fibrinogen (160–350 mg/dL)	294	Anti-zic4 antibody	(–)
D-dimer (0.00–0.50 µg/mL)	2.40	Anti-GAD65 antibody	(–)
		Anti-Tr antibody	(–)
Blood chemistry		Anti-NMDAR antibody [CBA]	(–)
Total protein (6.6–8.1 g/dL)	6.9	Anti-LGI1 antibody [CBA]	(–)
Albumin (4.1–5.1 g/dL)	3.9	Anti-Caspr2 antibody [CBA]	(–)
Total bilirubin (0.4–1.5 mg/dL)	1.2	Anti-AMPAR antibody [CBA]	(+)
Aspartate aminotransferase (13–30 U/L)	26	Anti-GABABR antibody [CBA]	(–)
Alanine Aminotransferase (10–42 U/L)	17	Anti-DPPX antibody [CBA]	(–)
Amylase (44–132 U/L)	97	Anti-GlyR antibody [CBA]	(–)
Alkaline phosphatase (38–113 U/L)	190	Anti-GABAAR antibody [CBA]	(–)
γ-glutamyltransferase (13–64 U/L)	29	Anti-MOG antibody [CBA]	(–)
Creatine kinase (59–248 U/L)	290	Anti-AQP4 antibody [CBA]	(–)
Lactate dehydrogenase (124–222 U/L)	198		
Urea nitrogen (3.7–7.0 mg/dL)	4.9	Cerebrospinal fluid	
Creatinine (0.65–1.07 mg/dL)	0.89	Pressure (70–180 mmH_2_O)	120
Sodium (138–145 mEq/L)	136	Cells (0–3/µL)	11
Potassium (3.6–4.8 mEq/L)	3.7	Lymphocytes (/µL)	10
Chlorine (101–108 mEq/L)	97	Neurophils (/µL)	1
Calcium (8.8–10.1 mg/dL)	9.2	Protein (10.0–40.0 mg/dL)	81.4
Phosphorus (2.7–4.6 mg/dL)	3.1	Immunoglobulin G index	0.49
Fasting glucose (73–109 mg/dL)	168	MBP (pg/mL)	114
C-reactive protein (<0.14 mg/dL)	0.32	OCB	(–)
sIL-2R (122–496 U/mL)	426	Glucose (50–75 mg/dL)	77
Immunoglobulin G (861.0–1747.0 mg/dL)	1399.9	sIL–2R (U/mL)	174.0
Immunoglobulin A (93.0–393.0 mg/dL)	136.8	IL–6 (pg/mL)	51.7
Immunoglobulin M (33.0–183.0 mg/dL)	99.8	Anti-NMDAR antibody [CBA]	(–)
Anti-nuclear antibodies (<40)	40	Anti-LGI1 antibody [CBA]	(–)
Rhumatoid factor (<15.0 IU/mL)	6.9	Anti-Caspr2 antibody [CBA]	(–)
Anti dsDNA antibody (<10.0 IU/mL)	0.9	Anti-AMPAR antibody [CBA]	(+)
Anti SS-A antibody (<7.0 U/mL)	<0.4	Anti-GABABR antibody [CBA]	(–)
Anti SS-B antibody (<7.0 U/mL)	<0.4	Anti-DPPX antibody [CBA]	(–)
MPO-ANCA (<0.2 IU/mL)	<0.2	Anti-GlyR antibody [CBA]	(–)
PR3-ANCA (<0.6 IU/mL)	<0.6	Anti-GABAAR antibody [CBA]	(–)
Free T4 (0.90–1.7 ng/dL)	1.35	Anti-MOG antibody [CBA]	(–)
Thyroid stimulating hormone (0.50–5.00 μIU/mL)	1.22	Anti-AQP4 antibody [CBA]	(–)
Anti‐thyroglobulin antibody (<28 IU/mL)	15.7	microbiological cultures	(–)
Anti-thyroid peroxidase antibody (<16 IU/mL)	11.7	Herpes simplex virus	(–)

AChR = Acetylcholine receptor, AMPAR = α-amino-3-hydroxy-5-methyl-4 isoxazolepropionic acid receptor, APTT = activated partial thromboplastin time, AQP4 = Aquaporin 4, Caspr2 = Contactin-associated protein 2, CBA = cell based assay, DPPX = Dipeptidyl aminopeptidase-like protein 6, dsDNA = double-stranded DNA, GABAAR = γ-amino butyric acid A receptor, GABABR = γ-amino butyric acid B receptor, GAD = Glutamate decarboxylase, GlyR = Glycine receptor, IL-6 = Interleukin-6, LGI1 = Leucine-rich glioma-inactivated protein 1, MBP = myelin basic protein, mGluR5 = Metabotropic glutamate receptor 5, MOG = Myelin oligodendrocyte glycoprotein, MPO-ANCA = myeloperoxidase-antineutrophil cytoplasmic antibody, NMDAR = N-methyl-D-aspartate receptor, OCB = oligoclonal band, PR3-ANCA = proteinase 3-antineutrophil cytoplasmic antibody, PTINR = prothrombin time-international normalization ratio, sIL-2R = soluble interleukin-2 receptor.

## 3. Discussion

AMPAR are critical mediators of rapid excitatory neurotransmission in the central nervous system and play an essential role in synaptic plasticity, learning, and memory.^[8]^ Accordingly, AMPAR antibody encephalitis typically presents with prominent cognitive impairment and psychiatric symptoms. AMPARs are widely expressed in the hippocampus, cerebellum, caudate nucleus, and cerebral cortex,^[9]^ and the associated encephalitis most commonly involves limbic and cortical regions. Clinically, the disorder has been classified into confused, epileptic, amnestic, and fulminant encephalitis.^[10]^ The present patient demonstrated prominent features of cortical encephalitis and followed a fulminant clinical course. Approximately 60% of patients with AMPAR antibody encephalitis have an associated tumor, most commonly thymoma, small-cell lung cancer, or breast cancer.^[11]^ Tumor-associated antigen expression and subsequent autoantibody production are considered central to disease pathogenesis. First-line immunotherapeutic strategies include high-dose glucocorticoids, intravenous immunoglobulin, plasma exchange, and combined immunotherapy.^[12]^ In patients with coexisting tumors, early tumor resection is recommended. Although immunotherapy is generally considered effective in AMPAR antibody encephalitis, patients with AMPAR antibody positivity tend to have a poorer overall prognosis and a higher risk of relapse compared with other forms of autoimmune encephalitis.^[13]^ In a previous study, 5 of 22 patients received rituximab; among these, two showed partial improvement, whereas three demonstrated no response, similar to the present patient. Miao et al reviewed 18 patients with thymoma-associated autoimmune encephalitis secondary to MG.^[14]^ In that review, 5 of 18 patients had AMPAR antibodies detected in serum and CSF. All patients tested positive for anti-AChR antibodies. Additionally, 12 of 18 patients showed a favorable response to thymectomy and immunotherapy. However, among the remaining six patients, one developed severe cognitive impairment and died. Therefore, rituximab-based immunotherapy may not be uniformly effective in patients with AMPAR antibody encephalitis associated with MG.

Five patients with g-TAMG with AMPAR antibody encephalitis have been reported (Table 2).^[12,14–17]^ Except for two patients in whom thymoma was identified at the onset of encephalitis, all patients, including the present patient, had undergone thymectomy. However, the clinical status of MG does not necessarily correlate with that of encephalitis. The reasons for this discrepancy may include differential responses of the two disorders to immunotherapy. Most patients have been reported to respond well to steroid pulse therapy with relatively favorable neurological outcomes. However, the present patient developed fulminant encephalitis with poor response to immunotherapy. The limited efficacy of rituximab may have been related to delayed administration or irreversible neuronal injury prior to treatment. According to our hospital’s regulations, the administration of rituximab must be reviewed by the ethics committee prior to administration. For this reason, early administration was difficult. In addition to the delayed administration, we also considered the possibility that the fulminant encephalitis phenotype had caused rapid neuronal damage, resulting in irreversible damage. Prognostic factors include the presence of tumors and coexisting autoantibodies. Although anti-collapsin response mediator protein 5 (CRMP5) antibodies are associated with rapid deterioration and fatal outcomes in some patients.^[18,19]^ Although anti-CRMP antibodies were negative in the present patient, anti-titin and anti-Yo antibodies were weakly positive. The anti-Yo result was inconsistent with the clinical course, suggesting a false-positive finding. Conversely, anti-titin antibodies are well-established antistriational antibodies associated with g-TAMG.

**Table 2:** Summary of 6 g-TAMG associated with AMPAR antibody encephalitis cases including ours.

Case	Authors	Age	Sex	Type of onset	Tumor	WHO classification	Recurrence of tumor	Other antibodies	Treatment	Outcome
1	Li et al 2015.^[15]^	47	f	Confusion	Thymoma (concurrent with first episode of encephalitis)	B1	-	AChR and Titin antibodies	IVMP, azathioprine, thymectomy	Mild anomic aphasia
2	Luo et al 2019.^[16]^	50	f	Amnestic	Thymoma treated with thymectomy	B2	None	AChR antibodies	IVMP, azathioprine	Mildlong-term memory deficit
3	Su M et al 2023.^[14]^	39	f	Confusion	Thymoma treated with thymectomy and radiation therapy	B1	None	AChR, NMDAR and titin antibodies	IVMP, IVIg	Returned to baseline
4	Tan et al 2024.^[17]^	43	f	Confusion	Thymoma treated with thymectomy and radiation therapy	–	None	AChR antibodies	IVMP, IVIg, PE	Returned to baseline
5	Huang J et al 2025.^[12]^	32	m	Amnestic	Thymoma (concurrent with first episode of encephalitis)	B3	–	AChR antibodies	IVMP, IVIg, thymectomy	Returned to baseline
6	Present case	53	m	Fulminant encephalitis	Thymoma treated with thymectomy and radiation therapy	B1/B2	None	AChR and Titin antibodies	IVMP, IVIg, PE, RTX	No treatment response

g-TAMG = Generalized thymoma-associated myasthenia gravis, AMPAR = Anti-α-amino-3-hydroxy-5-methyl-4 isoxazolepropionic acid receptor, WHO = World Health Organization, AChR = Acetylcholine receptor, NMDAR = N-methyl-D-aspartate receptor, IVMP = Intravenous methylprednisolone pulse, IVIg = Intravenous immunoglobulin, PE = Plasma exchange, RTX = Rituximab.

Thymomas are associated with various autoimmune diseases, likely reflecting impaired immune tolerance due to the production of self-reactive T cells. The elimination of autoreactive T cells depends on interactions between T cells and thymic epithelial cells within the thymus.^[20]^ In MG, thymic epithelial cells are implicated in disease pathogenesis, with an association reported particularly with type B thymomas.^[21]^ Among five previously reported patients with g-TAMG associated with AMPAR antibody encephalitis, thymoma histologies were type B1 in two, type B2 in one, and type B3 in one, whereas the histological subtype was not specified in the remaining patient. Further accumulation of data is required to improve understanding of the pathogenesis of organ-specific autoimmune diseases.

## 4. Conclusion

In conclusion, immunotherapy, including rituximab, may have limited efficacy in g-TAMG associated with AMPAR antibody encephalitis. Even post thymectomy, early recognition and testing for AMPAR antibodies in patients with new central nervous system symptoms and early initiation of immunotherapy should be considered.

## Author contributions

**Data curation:** Jun Soma.

**Formal analysis:** Jun Soma, Keiko Tanaka.

**Investigation:** Jun Soma.

**Conceptualization:** Jun Sawada.

**Supervision:** Jun Sawada, Keiko Tanaka, Naoki Nakagawa.

**Resources:** Keiko Tanaka.

**Writing – original draft:** Jun Soma.

**Writing – review & editing:** Jun Soma, Jun Sawada, Momoko Nanbu, Rei Ando, Shiori Kikuchi-Takeguchi, Keiko Tanaka, Naoki Nakagawa.
